# Seq-ing answers: exploring meningioma biology utilizing bulk RNA-seq-based reference landscapes

**DOI:** 10.3389/fonc.2025.1631573

**Published:** 2025-08-27

**Authors:** Abigail G. Parrish, Eric C. Holland

**Affiliations:** Human Biology Division, Fred Hutchinson Cancer Center, Seattle, WA, United States

**Keywords:** meningioma, bulk RNA-seq, reference landscape, UMAP, oncoscape

## Abstract

Meningiomas are the most common primary brain tumors, accounting for 40% of all central nervous system neoplasms. While usually benign, these tumors can vary in aggressiveness. Traditional classification and grading systems, which primarily rely on histopathological features, are not always reliable in capturing tumor behavior and predicting patient outcomes. In contrast, modern systems—based on factors such as copy number alterations, DNA methylation, and gene expression—offer a more accurate framework for identifying distinct biological signatures and aggressive subtypes, as well as for predicting recurrence. Transcriptomic profiling using bulk whole-genome RNA sequencing (RNA-seq), which provides insights into alternative splicing, gene expression, fusion events, non-coding RNAs, and pathway activity, further enhances our understanding of meningioma tumorigenesis, enables the projection of new samples onto dimension-reduced reference landscapes, and helps accurately predict recurrence. As bulk RNA-seq becomes more accessible, it holds great potential for refining prognostic tools, informing personalized treatment approaches, and ultimately improving outcomes for meningioma patients.

## 1 Introduction

### 1.1 Overview

With an incidence of 9.51 per 100,000 people, meningiomas are the most common primary central nervous system (CNS) tumor, comprising around 40% of all CNS tumors and approximately 55% of all non-malignant tumors (1). The incidence of meningiomas increases with age, with the median age at diagnosis being 67 years (1). These tumors develop in the meninges, the three layers of tissue beneath the skull that cover and protect the brain, and are thought to arise from the arachnoid cap cells in the middle meningeal layer, the arachnoid mater. Although most meningiomas are benign and slow-growing, often remaining undetected for years, a small subset has aggressive underlying biology, frequently recur, and can eventually be fatal.

### 1.2 Classification and grading systems based on histopathology

Meningiomas can present with a wide range of clinical manifestations, influenced by various factors. As our understanding of these tumors has advanced over time, their classification and grading systems have evolved to better reflect the insights we have gained. The term “meningioma” itself was coined by neurosurgeon and pathologist Harvey Cushing more than a century ago to describe tumors occurring along the neuraxis, replacing the term “dural endothelioma” (2). Over the next decade, Cushing, in collaboration with neuropathologist Louise Eisenhardt, published a monograph, providing a detailed classification, regional behavior, life history, and surgical outcomes of meningiomas, laying a foundation for the modern understanding and treatment of these tumors (3).

Building on this foundation, Australian neurosurgeon Donald Simpson introduced the Simpson grading scale in 1957, a system designed to use the extent of surgical resection completeness as a predictor of meningioma recurrence (4). Grades range from grades I through V, with grade I indicating macroscopic complete tumor resection with removal of affected dura and bone, and grade V indicating simple decompression with or without biopsy. While this grading scale primarily focused on the macroscopic extent of surgical resection, it did not account for the histopathological features of the tumors.

To address these limitations, the World Health Organization (WHO) established a new classification system in 1979, offering a more comprehensive framework based on microscopic analysis of tumor cells. Specifically, this system was primarily based on histological criteria such as mitotic rate and brain invasion, and categorized meningiomas into several subtypes based on histopathological architecture (i.e. meningothelial, fibrous, transitional, psammomatous, angiomatous, hemangioblastic, hemangiopericytotic, and papillary subtypes), along with an additional, more aggressive subtype known as anaplastic (5).

### 1.3 Advancements in molecular and genetic technology

Although the WHO classification system offered a more nuanced understanding of meningioma heterogeneity, aiding in prognosis and treatment planning, it did not fully address the molecular and genetic drivers underlying tumorigenesis. While the WHO classification system was being developed and implemented, chromosome 22q loss was identified as a recurrent genetic alteration in meningiomas (6). In the following decades, the inactivation of the NF2 tumor suppressor gene on chromosome 22, which acts as a master upstream regulator of the Hippo signaling pathway, was recognized as the most common genetic alteration in meningioma oncogenesis and as the genetic alteration responsible for the familial syndrome, neurofibromatosis type 2 (NF2). Loss of NF2 function allows YAP1 to remain active, translocating into the nucleus, binding to TEAD transcription factors, and driving cell survival, growth, and proliferation.

By the late 1990s and early 2000s, advances in genetic technology revealed additional genetic subtypes in NF2 wild-type meningiomas. Notable among these were mutations in genes such as TRAF7, AKT1, KLF4, PIK3CA, and SMO, which were identified between 2013 and 2016 (7–9). While mutations in AKT1, KLF4, and PIK3CA are generally mutually exclusive, they frequently co-occur with TRAF7 mutations (10). Taken together, the identification of a greater number of candidate mutations in meningiomas can be largely attributed to advancements in whole-genome and whole-exome sequencing.

### 1.4 Incorporating advancements into the WHO grading system

As our understanding of the biology of meningiomas has evolved, several molecular characteristics have become increasingly important in their classification and grading. Currently, the fifth edition of the 2021 WHO Classification of Tumors of the Central Nervous System (CNS5) stratifies meningiomas into three grades across several histopathologic subtypes: nine variants in WHO CNS5 grade 1, three in grade 2 (atypical, choroid, and clear cell), and two in grade 3 (papillary and rhabdoid) (5). CNS5 also incorporates high-risk molecular markers, including SMARCE1 in the clear cell subtype, BAP1 in the papillary and rhabdoid subtypes, and TERT promoter mutations and homozygous deletions of CDKN2A/B in grade 3 tumors (11, 12).

Brain invasion is now a standalone diagnostic criterion for atypical grade 2 meningiomas, regardless of other histological features. While tumor location is not a diagnostic criterion, high-grade meningiomas are more common in convexity and non-skull base areas, which often harbor chromosome 22q deletions and/or NF2 mutations. In contrast, skull base tumors are associated with mutations independent of NF2 (13–16). While certain molecular markers, brain invasion, and tumor location may define distinct and aggressive subclasses of meningiomas, their molecular and clinical roles are still poorly understood and warrant further investigation (17).

In response to these gaps in knowledge, the Consortium to Inform Molecular and Practical Approaches to CNS Tumor Taxonomy (cIMPACT-NOW) formed a working group to explore emerging molecular criteria. Their guidance has clarified when molecular testing should supplement morphological diagnosis and known genetic aberrations, and has been a major contribution to CNS tumor diagnostics (18).

Nevertheless, meningioma biology is nuanced, and although histopathology and genetic abnormalities provide valuable insights, they cannot fully account for the factors driving aggressive clinical behavior and unfavorable outcomes in certain patients. This disconnect has fueled growing interest in identifying molecular predictors of aggressiveness and redefining classification and grading systems accordingly.

### 1.5 Moving beyond traditional classification and grading systems

In recent years, several new grading systems based on copy number alterations, DNA methylation, and gene expression have been proposed, which offer more accurate prognostic tools. For instance, in 2019, a group from Baylor used RNA sequencing (RNA-seq) and whole-exome sequencing to identify three distinct molecular groups that correlated more closely with tumor recurrence than the 2016 WHO grading system: Group A (mutations in TRAF7, AKT1, and KLF4), Group B (loss of chromosome 22q), and Group C (loss of repressive DREAM complex function) (19). In 2021, researchers from the University of Toronto integrated DNA somatic copy number alterations, point mutations, DNA methylation, and messenger RNA abundance to identify four molecular subgroups: MG1 (immunogenic), MG2 (benign NF2 wild-type), MG3 (hypermetabolic), and MG4 (proliferative) (20). The following year, researchers from the University of California, San Francisco (UCSF) used DNA methylation profiling to identify three DNA methylation groups: merlin-intact, immune-enriched, and hypermitotic (21, 22). Around the same timeframe, the same UCSF lab published a 34-gene expression biomarker that is prognostic for clinical outcomes (23). While these molecular classification systems were more effective at identifying aggressive high-risk meningiomas than traditional histological classification systems, there is still a need for more comprehensive tools.

### 1.6 Application of artificial intelligence in meningioma research

Beyond molecular and genetics-based classification systems, recent advances in artificial intelligence (AI), particularly in machine learning, have significantly transformed how pathologists and radiologists classify meningiomas. These technologies offer a more detailed, objective understanding of tumor characteristics by integrating image-based data with clinical and molecular information, moving far beyond traditional visual assessments. One such innovation is radiomics, a subfield of AI that extracts quantitative features from medical imaging modalities such as CT, MRI, and PET scans. Radiomic models have shown promising results, with some studies reporting diagnostic accuracies as high as 93% when distinguishing grade 1 meningiomas from higher-grade (grades 2-3) tumors using structural MRI (24). However, tumor grade alone does not always predict clinical behavior, as some grade 1 tumors recur as rapidly as grade 3 tumors, while certain tumors classified as high-grade may follow a more indolent course. This highlights the limitations of relying solely on radiology and WHO grading to predict tumor aggressiveness and patient outcomes.

Building on this, emerging work in radiogenomics, which links radiomic features to molecular markers, genetic mutations, and chromosomal aberrations, has further enhanced our ability to preoperatively stratify meningiomas. For instance, meningiomas identified as high-risk based on imaging features have been significantly associated with molecular indicators of aggressive tumor biology, such as increased somatic mutation burden, altered DNA methylation patterns, and elevated expression of pro-mitotic transcription factors like FOXM1 (25). However, despite these correlations, some tumors that appear benign based on radiomic profiles may still behave aggressively, underscoring a critical insight: biological signatures often offer a more accurate prediction of patient outcomes than imaging, molecular, and genetic features.

AI is a very effective and powerful tool at answering specific questions, such as identifying tumor incidences or grade-gene associations, but currently, it cannot explore and discern complex data and reliably predict patient outcomes. To address this, we need more advanced tools that go beyond existing classification systems—tools capable of uncovering hidden patterns and clustering tumors based on shared biological signatures rather than grade or appearance. Only then can we develop a more accurate, predictive framework for understanding and managing tumor progression.

### 1.7 Utilizing bulk RNA-seq data to generate reference landscapes

While both traditional and modern classification systems, enhanced by advances in AI, provide valuable insights into tumor biology, they still fall short in fully capturing how specific biological signatures influence clinical outcomes. The aggressive nature of some tumors likely reflects their underlying biological characteristics, which are closely linked to gene expression patterns. Beyond simply measuring gene expression levels, RNA-seq captures additional complexities, such as allele-specific expression, alternative splicing, and fusion events, which can aid our understanding and identification of biomarkers, tumor heterogeneity and evolution, drug resistance, and so on. These molecular details reveal specific signatures associated with recurrence and treatment response—factors that may not be apparent through morphological assessments alone.

Since bulk RNA-seq is not limited by the number of cells, it can handle larger sample sizes and is suitable for studies requiring high throughput. Additionally, since it pools RNA from many cells, bulk RNA-seq can detect low-abundance transcripts more easily, providing a comprehensive overview of gene expression across sample populations.

**Figure 1:**
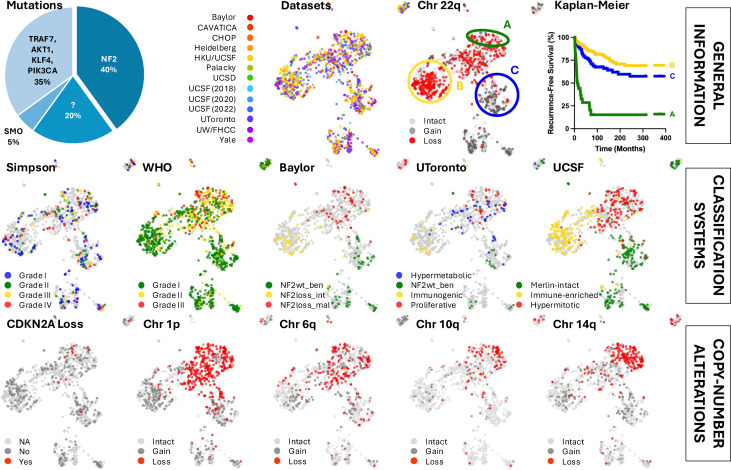
Various classification systems and copy-number alterations show regional patterns across the meningioma bulk RNA-seq-based reference landscape.

By integrating multiple publicly available human meningioma RNA-seq datasets that include 1298 tumors, the largest clinically annotated meningioma RNA-seq database and reference landscape has been created using uniform manifold approximation and projection (UMAP), complete with an interactive tool for further exploring tumor biology and signatures (**Figure 1**) (26). This comprehensive resource not only deepens our understanding of different meningioma subtypes and genetic signatures, but also enables a more precise approach to predicting their behavior.

## 2 Body

### 2.1 Reference landscape identifies regional associated biology

RNA sequencing is effective at detecting copy number alterations. Coloring the map for tumors with known chromosome 22q loss revealed distinct clustering of chromosome 22 and NF2 expression status. The UMAP analysis also identified several subtypes with particularly poor outcomes, with the most aggressive tumors enriched on one end of the NF2 mutant region, corroborating the well-established phenomenon that aggressive tumors frequently harbor NF2 mutations. These tumors exhibit high proliferation rates and upregulation of genes associated with embryonic limb development (26). Additionally, it has been shown that aggressive NF2 mutant meningiomas downregulate oncogenic YAP1 signaling. This downregulation occurs, at least in part, through the upregulation of YAP1 antagonist VGLL4 and the upstream regulator FAT4 (27).

Consistent with previous findings, malignant meningiomas often display one or more focal chromosomal deletions, with additional genetic mutations linked to accelerated growth and higher tumor grades. In 1998, Ishino and colleagues discovered a high incidence of partial deletion of chromosome 1p in atypical and anaplastic meningiomas (28). This deletion helps distinguish the more aggressive NF2 mutant meningiomas from their benign counterparts. Specific chromosomal mutations, including those on 1p, 6q, 10q, and 14q, have been implicated in meningioma malignancy and recurrence risk, while chromosome 9p loss and CDKN2A is also associated with malignant tumors and poorer prognosis (29–31). Notably, the aggressive region of the UMAP reveals losses on chromosomes 1p, 6q, 10q, and 14q, with most CDKN2A-null samples clustering in this area. Tumors with high levels of aneuploidy strongly correlated with shorter recurrence times compared to patients without these copy number alterations (26).

### 2.2 Investigating the role of copy number alterations in aggressive NF2 mutant meningiomas

It is well-established that the initiating events in other brain and central nervous system cancers often involve chromosomal nondisjunction, with tumor suppressors typically driving these arm-level genomic changes, such as the case of PTEN on chromosome 10q in glioblastoma (32). Similarly, it is both plausible and reasonable to consider that allelic losses on these chromosomes in meningiomas may also be driven by the loss of tumor suppressors, warranting further investigation. One promising approach is to analyze these chromosomal regions gene-by-gene, examining the correlations between chromosomal alterations, gene expression, and patient outcomes.

### 2.3 Mouse models of NF2 mutant-like meningiomas

While several cell line and patient-derived xenograft (PDX) mouse models of meningioma exist, reliable genetically engineered mouse models (GEMMs) that induce *de novo* meningioma formation have only recently been developed (33). Using the RCAS/tv-a system (RCAS = Replication-Competent Avian sarcoma-leukosis virus long terminal repeat with a Splice acceptor, tv-a = tumor virus receptor A), which helps facilitate a somatic gene transfer in transgenic mice, researchers have demonstrated that the exogenous expression of constitutively active YAP1 is sufficient to induce NF2 mutant-like meningiomas in mice (34). By identifying strong oncogenic drivers in meningiomas, researchers can leverage the RCAS/tv-a system and other GEMMs to establish causality. More specifically, the RCAS/tv-a system allows for the short hairpin RNA-mediated knockdown or conditional knockout of specific tumor suppressor genes located on these chromosomes of interest, enabling the study of tumor suppressor losses and their effects on tumor latency, biology, and treatment response.

## 3 Discussion

### 3.1 Gaining novel insights from bulk RNA-seq-based reference landscapes

Historically, classification and grading systems in fields like histopathology were shaped by the tools and knowledge available at the time, with early methods relying heavily on observable characteristics. As technology has advanced, we have gained powerful tools that allow us to delve deeper into the underlying biology of diseases, enhancing our ability to understand and accurately predict patient outcomes. One such tool is dimension-reduced reference landscapes, which are invaluable for visualizing complex biological data. By condensing high-dimensional data, such as gene expression and clinical metadata, into a more manageable, lower-dimensional space, algorithms like UMAP enable researchers to identify patterns, clusters, relationships, and potential prognostic factors within vast datasets. This visualization helps uncover subtle differences between tumor subtypes, revealing variations in aggressiveness, treatment response, and overall survival. By integrating UMAP with existing classification systems, researchers can not only refine current models but also gain novel insights that could lead to a better understanding of diseases and more personalized treatment approaches.

### 3.2 Accessibility and limitations of bulk RNA-seq

Advances in technology, such as the ability to effectively extract high-quality RNA from formalin-fixed paraffin-embedded (FFPE) samples and improved multiplexing, have made RNA-seq more accessible and an invaluable tool for both research and clinical applications. Assuming an optimal batch size and a fully dedicated team, the entire workflow—including sectioning, RNA isolation, library preparation, sequencing, and bioinformatic analysis (with a validated pipeline in place)—can be completed within 7 to 10 days at a cost of a few hundred dollars per sample. This price has steadily declined over time, and this trend is projected to continue.

While cost-effective and high-throughput, bulk RNA-seq averages gene expression across a sample, failing to capture the diversity of individual cell types and their specific contributions. Further, bulk RNA-seq cannot differentiate between individual cells, making it difficult to detect rare cell populations and more subtle changes in gene expression over time, and lacks spatial context, preventing the identification of a cell’s location within the sample. Alternatively, while more expensive, single-cell RNA-seq (scRNA-seq) allows for the investigation of gene expression at the individual cell level, making it possible to study cellular heterogeneity and discover rare cell types and states that might be masked by bulk RNA-seq. Similarly to bulk RNA-seq data, scRNA-seq data can also be mapped onto reference landscapes for analytical purposes.

### 3.3 The clinical impact of bulk RNA-seq-based reference landscapes

As bulk RNA-seq data becomes increasingly available, it offers powerful potential to identify biological signatures that predict outcomes and guide personalized therapies. Expanding RNA-seq databases will be instrumental in advancing precision medicine and optimizing care across disease and tumor types.

One of the most powerful applications of bulk RNA-seq is mapping patients onto interactive reference maps that integrate gene expression and clinical data. For instance, consider the case of Mrs. Smith, a hypothetical patient who is diagnosed with a WHO grade 1 meningioma and undergoes surgical resection. Initially, her prognosis appears favorable. However, within a year, the tumor recurs, once again classified as a grade 1 tumor. In the past, this recurrence would leave clinicians grappling with uncertainty: was the tumor not fully resected, or is this a sign of inherently more aggressive underlying biology?

**Figure 2:**
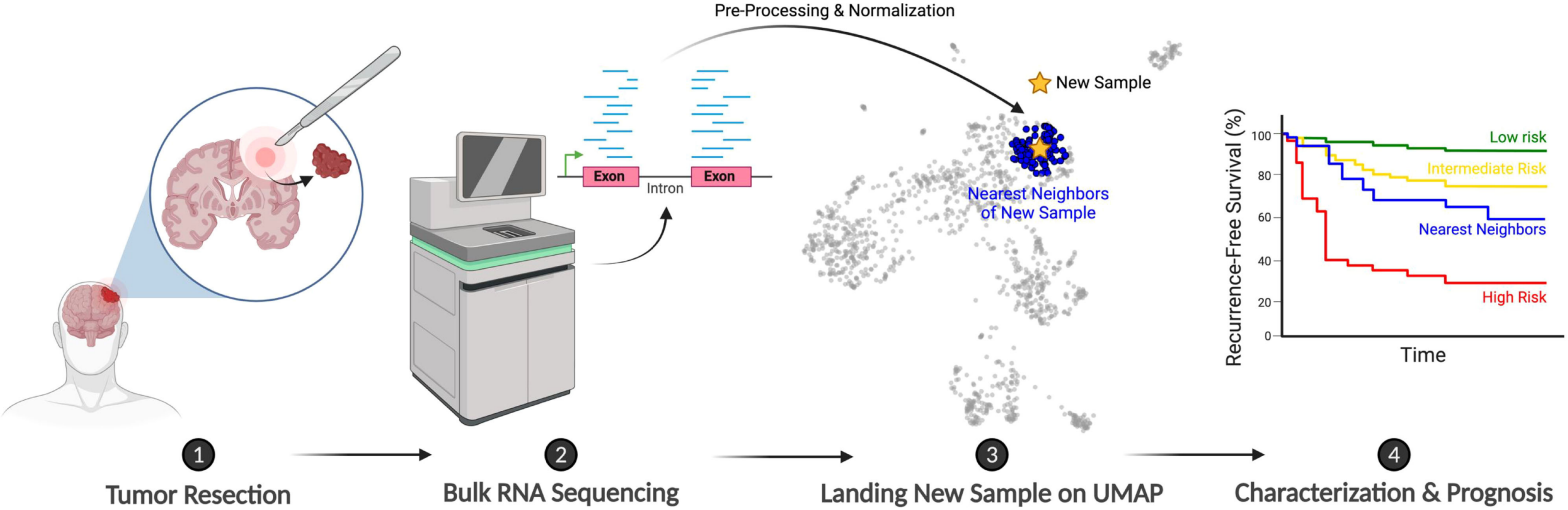
Schematic illustrating the proposed clinical workflow for integrating new patients onto the meningioma bulk RNA-seq-based reference landscape. Created with BioRender.com.

With RNA-seq-based reference landscapes, we no longer have to rely solely on guesswork. By positioning Mrs. Smith’s molecular profile within this map, clinicians can compare her case to those of similar tumors, her “nearest neighbors” (**Figure 2**). These comparisons can uncover patterns in tumor behavior, reveal the likelihood of recurrence, and even suggest specific therapeutic interventions, moving us closer to establishing RNA-seq as a routine tool in clinical decision-making.
